# Differential p38-dependent signalling in response to cellular stress and mitogenic stimulation in fibroblasts

**DOI:** 10.1186/1478-811X-10-6

**Published:** 2012-03-09

**Authors:** Dagmar Faust, Christina Schmitt, Franz Oesch, Barbara Oesch-Bartlomowicz, Ilona Schreck, Carsten Weiss, Cornelia Dietrich

**Affiliations:** 1 Institute of Toxicology, Medical Center of the Johannes Gutenberg-University, Obere Zahlbacherstr. 67, 55131 Mainz, Germany; 2 Institute of Toxicology and Genetics, Karlsruhe Institute of Technology, Campus North, Hermann-von-Helmholtz-Platz 1, Eggenstein-Leopoldshafen, Germany

**Keywords:** p38 MAPK, Signalling, Cellular stress, Mitogens, Fibroblasts

## Abstract

p38 MAP kinase is known to be activated by cellular stress finally leading to cell cycle arrest or apoptosis. Furthermore, a tumour suppressor role of p38 MAPK has been proposed. In contrast, a requirement of p38 for proliferation has also been described. To clarify this paradox, we investigated *stress*- and *mitogen*-induced p38 signalling in the same cell type using fibroblasts. We demonstrate that - in the same cell line - p38 is activated by mitogens or cellular stress, but p38-dependent signalling is different. Exposure to cellular stress, such as anisomycin, leads to a strong and persistent p38 activation independent of GTPases. As a result, MK2 and downstream the transcription factor CREB are phosphorylated. In contrast, mitogenic stimulation results in a weaker and transient p38 activation, which upstream involves small GTPases and is required for cyclin D1 induction. Consequently, the retinoblastoma protein is phosphorylated and allows G1/S transition. Our data suggest a dual role of p38 and indicate that the level and/or duration of p38 activation determines the cellular response, i.e either proliferation or cell cycle arrest.

## Background

The family of mitogen-activated protein kinases (MAPKs) involves ERKs (extracellular signal-regulated kinases), JNKs (c-Jun-N-terminal kinases) and p38 MAPKs. They are proline-directed Ser/Thr kinases mediating a variety of cellular responses due to numerous extracellular stimuli. While the ERK pathway is preferentially induced by mitogens, p38 and JNK are generally activated by inflammatory cytokines and cellular stress, such as hyperosmolarity, heat shock, genotoxic compounds, UV light, γ-irradiation, metabolic stress, and protein synthesis inhibition [for review see [1-4]]. Mammalian p38 was first cloned by Han and coworkers and revealed close homology to the yeast osmosensing HOG1 [5]. A common response of p38 activation by cellular stress is cell cycle arrest or apoptosis. Moreover and consistent with its role in cell cycle regulation, p38 is involved in oncogene-induced senescence, replicative senescence, differentiation, and DNA-damage response [for review see [6]]. For instance, several reports indicate an inhibitory role of p38 in proliferation in the mouse fibroblast cell line NIH3T3. Tunicamycin-treatment causes activation of p38 resulting in phosphorylation of GADD153 finally leading to cell cycle block [7]. G1-arrest is also detected in response to arsenite, which is mediated by a p38-dependent increase in p21 [8]. Constitutive expression of oncogenic ras results in sustained activation of p38 and inhibition of proliferation [9]. Consistent with a negative role in cell cycle progression, expression of active MKK3/6 induces G1 arrest in the same cell line [10]. Similar data has been obtained in rhabdomyosarcoma cells, in which p38 activation results in G1-arrest and differentiation [11]. We recently revealed that sustained activation of p38 in response to high cell density is involved in the signalling cascade of contact-inhibition in murine and in human FH109 fibroblasts by regulating levels of the Cdk-inhibitor p27 [12].

However, several reports suggest that p38 is also activated in mitogenic pathways. Maher described a requirement of p38 for bFGF-induced (but not for PDGF-stimulated) cell proliferation in Swiss3T3 fibroblasts [13]. A similar role has been proposed in hepatocytes after EGF- and insulin-treatment [14], and ERK cooperates with p38 in G-CSF-stimulated hematopoietic cell proliferation [15]. A proliferative role of p38 has also been described in breast cancer-, chondrosarcoma-, and melanoma cells [16-18]. These reports suggest a proliferative function of p38 in contrast to the above mentioned role of p38 in stress response and cell cycle arrest.

Some simple explanations for these discrepancies could be dependence on stimulus, cellular context or cell-type specificity. For example, p38 activation by one type of stimulus might lead to different biological outcomes, i.e. proliferation or growth arrest, dependent on cell type. Alternatively, distinct stimuli could induce p38 signalling via different routes in the very same cell type thus triggering diverging responses. Unfortunately, the proliferative and growth inhibitory role of p38 has been investigated so far only in different cell lines and thus the possibility of divergent p38 signalling downstream of different stimuli in simply the same cell line is poorly understood. Therefore we studied p38 signalling in human and murine fibroblast cell lines and compared the signalling cascades up- and downstream of p38 in response to mitogens and cellular stress. Here we show that mitogen-induced proliferation in NIH3T3 and FH109 fibroblasts can be substantially blocked by the compound SB203580, which is a selective inhibitor of p38 [19] again demonstrating that p38 mediates cellular proliferation. Since p38 also regulates cell cycle arrest in the same cell lines as mentioned above, we analysed mitogen- and stress-induced activation of p38 in NIH3T3 and FH109 fibroblasts. We provide evidence for a dual role of p38 in cell cycle control and suggest that the level and/or the duration of p38 activation might determine the cell's decision to proliferate or to induce cell cycle arrest. We show that in NIH3T3 and FH109 fibroblast cell lines mitogenic stimuli lead to a weak and transient phosphorylation of p38, which is absolutely required for G1/S-transition whereas anisomycin induces a strong and sustained activation of p38. We further revealed that the signalling cascades involving p38 activation after serum- and stress-treatment differ. While mitogenic activation of p38 upstream involves small GTPases and downstream leads to cyclin D1 expression, anisomycin-dependent activation of p38 is independent of GTPases and leads to phosphorylation of MK2 and finally CREB. In conclusion, our data provide an example of differentially wired p38 signalling in response to distinct stimuli, which results in specific outputs such as stress-induced cell cycle arrest and mitogen-induced proliferation.

## Results

### Involvement of p38 in mitogen-induced proliferation

**Figure 1:**
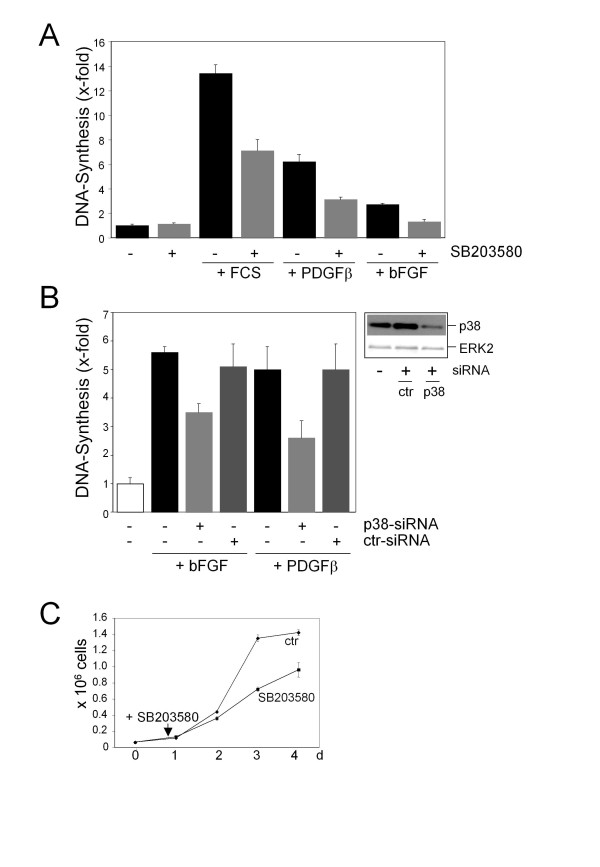
**Effect of impairment of p38 function on mitogen-induced DNA-synthesis. (A) **Serum-starved NIH3T3 cells were either not pretreated or preincubated for 1 h with SB203580 (10 μM). The cells were then stimulated with 10% FCS, PDGFβ or bFGF (each 50 ng/ml) for 20 h and labelled with [^3^H]thymidine for additional 4 h in the absence or presence of SB203580. Incorporated radioactivity was determined by liquid scintillation spectrometry. Results are given as x-fold induction of [^3^H]thymidine incorporation compared to unstimulated, serum-starved cells and are the average ± Sx of a number of four for each run of treatment. The results represent one out of three independent experiments each leading to similar results. **(B) **NIH3T3 cells were transiently transfected with siRNA directed against p38α or with control siRNA. Knock-down is shown by Western blot analysis. Cells were serum-starved and then stimulated with bFGF or PDGFβ. DNA-synthesis was determined as described in (A). (C) 0.06 × 10^6 ^NIH3T3 cells were seeded in 4.5 cm^2 ^dishes. Cells were either not treated or treated with SB203580 (10 μM) 4 h after seeding and then cultured in the absence or presence of SB203580 for another 68 h. Cells were counted in a hemocytometer. The results are expressed as the average ± Sx of a number of four for each time point and treatment. The results represent one out of two independent experiments.

Cellular stress is known to induce p38-dependent cell cycle arrest in NIH3T3 cells as does constitutive activation of p38 as mentioned above [7-10]. To investigate whether p38 is also involved in cell cycle progression, NIH3T3 were rendered quiescent by serum-depletion and then stimulated with fetal calf serum (FCS), platelet-derived growth factor-β (PDGFβ) or basic fibroblast growth factor (bFGF) in the absence or presence of the p38-specific inhibitor SB203580 [19] or after p38α-knock-down via transient transfection of siRNA targeted against murine p38α. p38α is the dominant isoform in NIH3T3 and FH109 fibroblasts [[20], and own unpublished observations]. As expected, stimulation with FCS or the growth factors resulted in a marked increase in DNA-synthesis as determined by [^3^H]thymidine incorporation. This increase was substantially blocked by SB203580, which was added one hour before the mitogens and was then present throughout the entire experiment, or after p38α knock-down (Figure 1A, B). Similar results were obtained in the human fibroblast cell line FH109 (Additional file 1). As stated below using the transcription factor CREB as a substrate, we demonstrated that SB203580 was indeed active for at least 24 h as a p38 inhibitor. Furthermore, we confirmed, that siRNA-mediated knock-down of p38α decreases growth factor-induced p38 phosphorylation. Importantly, phosphorylation of ERK1/2 was not affected up to 4 h (Additional file 2A, B). In line with the knock-down experiments, also the p38-inhibitor SB203580 did not alter mitogen-induced ERK1/2 phosphorylation at early time points, but decreased ERK1/2 phosphorylation at later time points (Additional file 2C). These data indicate that SB203580 does not directly inhibit ERK1/2 function, but that p38 activity seems to be required for sustained ERK1/2 phosphorylation. Although SB203580 is considered to be a highly specific inhibitor of p38 [19], inhibition of JNK2 has been described [21]. Here, phosphorylation of the JNK substrate c-Jun in response to anisomycin, a well-known activator of JNK, is not inhibited by SB203580, but as expected by the JNK-inhibitor SP600125 (Additional file 2D). Taken together, the previous experiments show that p38 is activated after mitogenic stimuli and is required for growth factor-induced re-entry of quiescent fibroblasts into the cell cycle.

To investigate whether p38 is also required for continuous proliferation, cells were seeded and cultured in 10% FCS up to 72 h in the absence or presence of SB203580. Figure 1C clearly shows that continuous proliferation of NIH3T3 cells is impaired in the presence of SB203580.

### Activation of p38 after mitogenic stimuli and cellular stress

**Figure 2:**
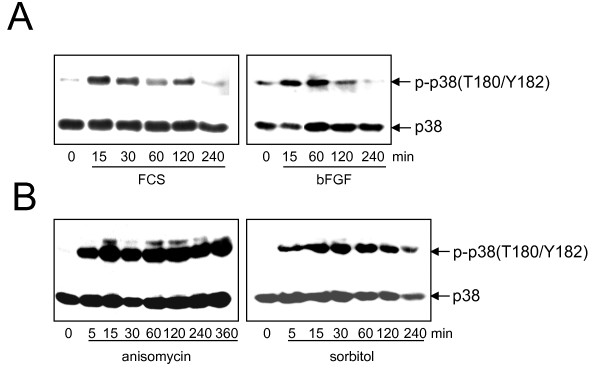
**Stimulation of p38 by mitogens and cellular stress. (A, B) **Serum-starved NIH3T3 cells were treated with FCS or bFGF **(A)**, anisomycin (10 &#956g/ml) or sorbitol (400 mM) **(B)** and total cell extracts prepared at the indicated time points. Western blot analysis was performed using a phospho-specific (T180/Y182) anti-p38-antibody. The blots were stripped and reprobed with an anti-p38-antibody to control equal loading. Data from a single experiment are shown. Similar results were obtained in three independent experiments.

p38 needs to be activated by dual phosphorylation on threonine (180) and tyrosine (182) by MKK3 or MKK6 [22]. We therefore made use of a phospho-specific anti-p38-antibody (T180/Y182) and performed Western blot analysis to compare p38 kinase activation in response to mitogens and cellular stress. Indeed, time course analysis revealed that p38 was clearly, but transiently phosphorylated after FCS- and bFGF-stimulation in serum-starved NIH3T3 cells (Figure 2A). In contrast, treatment with the cellular stressors and known inducers of p38 anisomycin or sorbitol [for review see [23]], results in a stronger and persistent phosphorylation of p38 (Figure 2B). Similar results were obtained in FH109 fibroblasts (Additional file 3).

### Cell cycle proteins are differentially affected by p38 signalling after mitogenic stimuli or cellular stress

**Figure 3:**
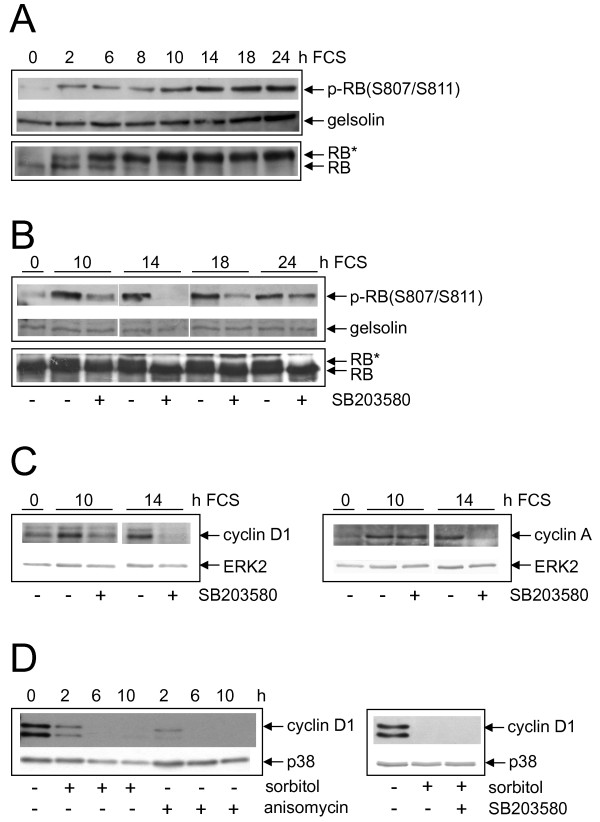
**Analysis of cell cycle proteins as potential p38 downstream targets. (A) **Serum-starved NIH3T3 cells were stimulated with FCS or **(B)** stimulated with FCS in the absence or presence of SB203580 for the indicated time points. Western blot analysis was performed using a phospho-specific (S807/S811) anti-pRB-antibody. The blots were stripped and reprobed with anti-gelsolin-antibody to control equal loading. The same samples were loaded and subjected to Western blot analysis using a pRB-antibody. RB* = phosphorylated pRB. **(C)** Serum-starved NIH3T3 cells were not pretreated or pretreated with SB203580 and then stimulated with FCS for the indicated time points. Western blot analysis was performed using anti-cyclin D1- (left) or anti-cyclin A-antibodies (right). **(D)** Serum-starved NIH3T3 cells were exposed to anisomycin or sorbitol for the indicated time points (left) or exposed to sorbitol for 6 h after preincubation with SB203580 (right). Western blot analysis was performed using anti-cyclin D1-antibody. Equal loading was controlled by stripping and reprobing the blots with anti-ERK2-antibody.

We next investigated cell cycle proteins as potential p38 downstream targets by Western blot analysis. A key regulator of G1/S-transition is the retinoblastoma protein (pRB). When phosphorylated, pRB dissociates from the transcription factor E2F thereby allowing transcription of S-phase specific genes, such as cyclin A, and subsequently entry into S-phase [24]. Using a phospho-specific anti-pRB-antibody we detected - as expected - phosphorylation of pRB after stimulation with FCS which reached maximal levels after 14 h and persisted thereafter (Figure 3A). Phosphorylation of pRB was also observed by a shift in the electrophoretic mobility of pRB. Importantly, preincubation with SB203580 prevents pRB phosphorylation (Figure 3B) indicating that p38 is required for S-phase entry. Consistent with the inhibition of pRB phosphorylation, maintained serum-induced expression of cyclin A was also abolished in the presence of SB203580 (Figure 3C).

Phosphorylation of pRB is mediated by cyclin-dependent kinases which are regulated by cyclins, namely by the Cdk4/cyclin D1 complex and the Cdk2/cyclin E complex. Hence, downregulation of cyclin D1 will lead to impaired phosphorylation of pRB. Indeed, serum-induced expression of cyclin D1 is strongly attenuated in the presence of SB203580 (Figure 3C). Downregulation of cyclin D1 expression by SB203580 was still detected 24 h after mitogenic stimulation (Additional file 4A). Thus, inhibition of p38 does not merely delay cyclin D1 expression, but prevents cyclin D1 induction. Similar findings were obtained in FH109 cells (Additional file 4B).

In contrast, anisomycin and sorbitol treatment led to a complete loss of cyclin D1 expression. However, this effect could not be reversed by SB203580 (Figure 3D). Hence, downregulation of cyclin D1 in response to cellular stress is probably mediated in addition by other stress kinases or pathways in NIH3T3 cells.

### Transcription factors are differentially targeted by p38 after mitogenic stimuli or cellular stress

**Figure 4:**
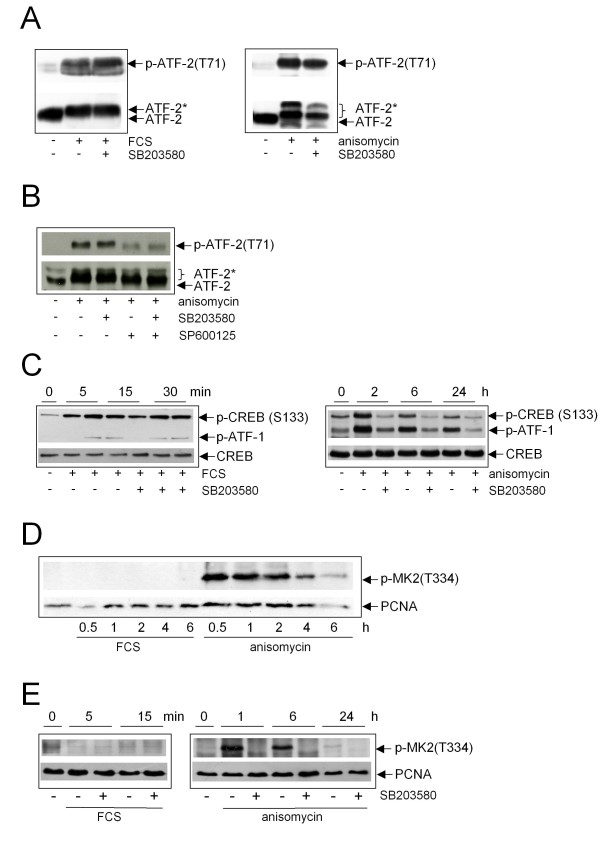
**Analysis of p38-mediated substrate phosphorylation. (A)** Serum-starved NIH3T3 cells were either not pretreated or pretreated with SB203580. Cells were not stimulated, stimulated with FCS (left) or with anisomycin (right) for 15 min. Total cell extracts were performed and subjected to Western blot analysis. Phosphorylation of ATF-2 was detected using a phospho-specific antibody, equal loading was controlled by stripping and reprobing the blot with anti-ATF-2-antibody. **(B)** NIH3T3 cells were either not treated or treated with anisomycin in the absence or presence of SB203580 (10 &#956M), SP600125 (25 &#956M) or both inhibitors for 15 min. Western blot analysis was performed as described above. **(C)** Serum-starved NIH3T3 cells were either not pretreated or pretreated with SB203580 and then stimulated with FCS (left) or anisomycin (right) for the indicated time points. Total cell extracts were performed and subjected to Western blot analysis. Phosphorylation of CREB was detected using a phospho-specific antibody, equal loading was controlled by stripping and reprobing the blot with anti-CREB-antibody. The anti-phospho-CREB-antibody also recognizes phosphorylated ATF-1. ATF-2* = phosphorylated ATF-2. **(D)** Serum-starved NIH3T3 cells were either not treated or treated with FCS or anisomycin for the indicated time points. Western blot analysis was performed using a phospho-specific anti-MK2-antibody. The blot was stripped and reprobed with anti-PCNA-antibody to control equal loading. **(E)** Serum-starved NIH3T3 cells were either not treated or treated with FCS or anisomycin for the indicated time points in the absence or presence of SB203580 (10 &#956M). Western blot analysis was performed using a phospho-specific anti-MK2-antibody. The blot was stripped and reprobed with anti-PCNA-antibody to control equal loading.

We next focussed our interest on known transcription factors. In co-transfection and *in vitro *experiments, the transcription factors activating transcription factor-2 (ATF-2) and cAMP-responsive element-binding protein (CREB) have been described to be excellent p38 substrates [25-27]. We therefore performed Western blot analysis with phospho-specific anti-ATF-2- and anti-CREB-(also recognizing ATF-1) antibodies after FCS- and anisomycin-exposure in the absence or presence of SB203580. To our surprise, ATF-2 phosphorylation was not inhibited by SB203580 neither after FCS- nor after anisomycin-treatment (Figure 4A). In contrast, phosphorylation of ATF-2 was strongly blocked by the JNK-inhibitor SP600125. Co-treatment of cells with both inhibitors of JNK and p38 did not further reduce ATF-2 phosphorylation demonstrating that JNK is the responsible kinase for ATF-2 phosphorylation in response to anisomycin (Figure 4B). These results argue against a requirement for p38 in ATF-2 phosphorylation in fibroblasts. Interestingly, also CREB phosphorylation in response to FCS-stimulation was not affected by SB203580 ruling out an involvement of p38 (Figure 4C). In contrast, anisomycin-induced phosphorylation of CREB/ATF-1 was totally abolished in the presence of SB203580 even after 24 h (Figure 4C) indicating that p38 is the only kinase mediating CREB phosphorylation under these conditions. Similar results were obtained in FH109 cells (Additional file 5). Consistent with these data, phosphorylation of MK2, which is known to mediate p38-dependent CREB phosphorylation [27,28], was only detectable after anisomycin-treatment, but not after FCS-stimulation, neither after prolonged treatment (Figure 4D), nor when analysing very early time points (Figure 4E). Anisomycin-induced CREB phosphorylation is mediated by the p38-MK2 pathway, as phosphorylation of MK2 in response to anisomycin was totally abolished in the presence of SB203580.

### FCS-, but not anisomycin-induced phosphorylation of p38 is dependent on GTPases

**Figure 5:**
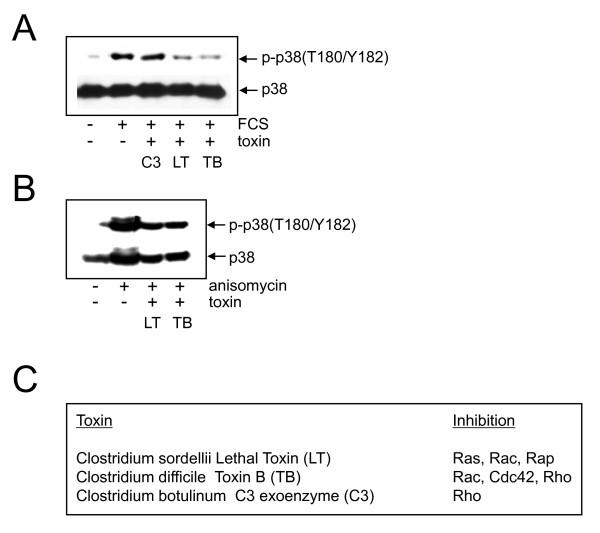
**Effect of inhibiting small GTPases by bacterial toxins on p38 phosphorylation. (A, B) **Serum-starved NIH3T3 cells were either not pretreated or preincubated for 23 h with 400 ng/ml of C2IN-C3 (C3), for 2 h with 200 ng/ml of Lethal Toxin (LT), or for 2.5 h with 10 ng/ml of Toxin B (TB). Cells were either not stimulated or stimulated for 30 min with FCS **(A) **or anisomycin **(B)**. Western blot analysis was performed with a phospho-specific anti-p38-antibody followed by reprobing with anti-p38-antibody. **(C) **Presentation of the bacterial toxins used indicating their substrate specificity according to [29-31].

We next investigated the involvement of small GTPases, comprising Ras, Rap and the family of Rho proteins (Rac, Rho and Cdc42) in p38 activation. Cells were preincubated with bacterial toxins to inhibit GTPase activity [for review see [29-31]]. Effective concentrations of the bacterial toxins were prior titrated by visualising changes in the integrity of the cytoskeleton via FITC-phalloidin staining (see addional file 6A). After preincubation with the toxins, cells were stimulated with FCS or anisomycin for 30 min and Western blot analysis was then performed with the phospho-specific anti-p38-antibody. Figure 5A clearly demonstrates that pretreatment with Lethal Toxin (LT) and Toxin B (TB), but not with C2IN-C3 toxin (C3) totally abolished serum-stimulated p38 phosphorylation. This indicates that FCS-dependent phosphorylation of p38 is dependent on small GTPases, but probably independent of Rho itself. None of the toxins (LT, TB, C3) was able to block anisomycin-induced p38 phosphorylation (Figure 5B). The specificity of the toxins was further demonstrated by the fact that ERK phosphorylation in response to FCS, which is downstream of Ras but not the other GTPases, was only blocked by LT but not by TB and C3 (Additional file 6B) [32]. The same was demonstrated in FH109 cells (data not shown). In conclusion, our results strongly argue for an involvement of GTPases in FCS-, but not in anisomycin-induced p38 phosphorylation.

**Figure 6:**
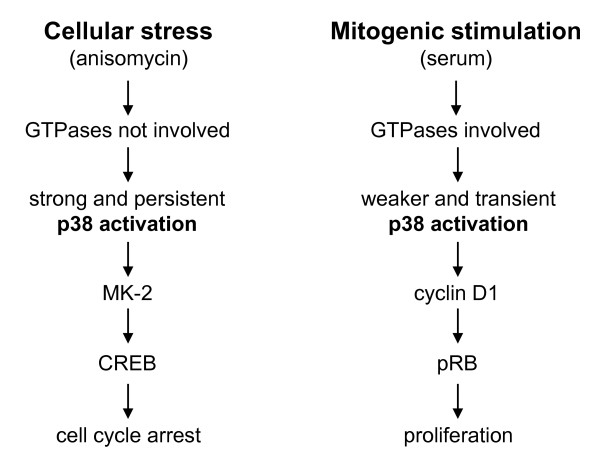
**Proposed hypothetical model describing different p38 signalling pathways in response to mitogens and cellular stress**. Anisomycin leads to a sustained phosphorylation of p38 which is independent of upstream-acting small GTPases and accompanied by an accumulation of p38 in the nucleus. One downstream substrate of p38 is CREB. In contrast, p38 is transiently phosphorylated after serum-stimulation in a GTPase-dependent manner and cyclin D1 is upregulated. The cellular response is proliferation.

In summary, we propose a hypothetical model for p38 signalling downstream of distinct stimuli which is depicted in Figure 6. We propose that discrete mechanisms lead to p38 activation after FCS- and anisomycin-exposure. Mitogens, but not cellular stress mediated by anisomycin, operate via small GTPases and subsequently trigger p38-dependent regulation of the retinoblastoma pathway. However, stress-induced p38 signalling initiates MK2 activity and hence phosphorylation of CREB without a concomitant proliferative response.

## Discussion

In the present work we show that p38 is activated by mitogens and cellular stress in the same cell line, but that the signalling pathways differ. We suggest that p38 plays a dual role in cell cycle control in fibroblasts mediating cell cycle progression or cell cycle arrest depending on the extracellular stimulus. In NIH3T3 cells, p38-dependent cell cycle arrest either due to cellular stress or constitutive activation by overexpression of the kinase itself or an upstream activating kinase has been demonstrated in various publications [7-10]. The fact that in the present study in the same cell line stimulation with serum or growth factors results in phosphorylation and activation of p38, and - vice versa - FCS- and growth factor-induced DNA-synthesis is blocked by the p38-specific inhibitor SB203580 or siRNA-mediated knock-down of p38 clearly indicates that p38 is also required for proliferation and points to a dual role of p38 in cell cycle regulation. Our data suggest that a key element might be the duration and/or amount of activation which then leads to different downstream signalling. While anisomycin-exposure leads to a strong and sustained activation of the p38 and MK2 kinases, thereby increasing the phosphorylation of CREB [27,28], mitogen-induced activation of p38 is weaker and transient, but is required for cyclin D1 expression. In addition, we demonstrate that p38 is differentially regulated in response to anisomycin and mitogens with respect to the involvement of small GTPases (Figure 6).

Dual regulation of a kinase depending on the extracellular stimulus has been proposed in the neuronal cell line PC12 for ERK [for review see [33]]. In these cells, EGF leads to a transient activation of ERK with cytoplasmic retention resulting in a proliferative cellular response. In contrast, NGF-induced ERK activation in the same cells is sustained and accompanied by a nuclear translocation causing cell cycle arrest and neuronal differentiation.

To better understand the role of p38 in mitogen-induced proliferation, we studied cell cycle proteins, i.e. phosphorylation of pRB and expression of the cyclins D1 and A. It is generally accepted that during G1-phase, cyclin D1/Cdk4 and downstream cyclin E/Cdk2 phosphorylate pRB which then dissociates from the transcription factor E2F allowing transcription of S-phase specific genes, such as cyclin A, and thereby entry into S-phase [24]. Since the mitogen-induced expression of cyclin D1 was strongly reduced in the presence of SB203580 we conclude that expression of cyclin D1 requires the activity of p38. An expected consequence of a decrease in cyclin D1 is less activity of the cyclin D1/Cdk4 complex and in turn less phosphorylation of pRB. Hence, the observed attenuation of pRB phosphorylation in the presence of SB203580 is very likely due to decreased cyclin D1 expression. Downregulation of cyclin A by SB203580 was observed at a later time point, i.e. 14 h after mitogenic stimulation. According to the kinetics of pRB phosphorylation we assume that this time point correlates with early S-phase. We therefore conclude that the decrease in cyclin A is not directly mediated by p38, but rather a consequence of inhibition of pRB phosphorylation by SB203580.

The mechanism of p38-dependent expression of cyclin D1 in our cell system is not known so far. In melanoma cells, p38-ATF-2-dependent expression of cyclin D1 in response to hepatocyte growth factor/scatter factor has been described [18]. Induction of cyclin D1 by pp60^v-*src *^is also mediated via the p38/JNK-ATF-2/CREB pathway in human breast cancer cells [34]. Since we did not detect p38-dependent phosphorylation of ATF-2 nor CREB, an involvement of ATF-2 or CREB in cyclin D1 expression in our cell system is unlikely. One possible explanation comes from the observation, that ERK1/2 phosphorylation is blocked from 6 h on after FCS-stimulation, very likely as a secondary effect of p38 inhibition. Hence, p38 activity seems to be required for sustained ERK1/2 phosphorylation. In fibroblasts, sustained ERK1/2 activity is required for cyclin D1 expression, especially during mid-G1-phase [35]. The underlying mechanism of this cross-talk between p38 and ERK1/2 remains to be elucidated.

To our surprise, inhibition of p38 function by SB203580 did not only block mitogen-induced G0/G1-S transition, but also attenuated continuous proliferation of NIH3T3 cells. This observation is in contrast to data obtained in BJ primary fibroblasts and WI-38 fibroblasts. In these cells, SB203580 does not alter proliferation in exponentially growing cultures, which show doubling rates comparable to our NIH3T3 [36,37]. However, cell type specific differences might explain different functions of p38 in NIH3T3 cells. Very recently, it was shown that the transcription factor FoxM1 acts downstream from the Ras-MKK3-p38 pathway in NIH3T3 cells [38]. Importantly, FoxM1 is also known to regulate a number of proliferative genes [38; manuscript in preparation]. Although we have not tested, another explanation for the discrepancies could be p53 function. While BJ and WI-38 fibroblast express wild-type p53 [39,40], the NIH3T3 cells we used are p53-deficient (unpublished observations).

Our observation of a sustained p38 activation after anisomycin- or sorbitol-treatment is in accordance with other reports showing persistent activation of p38 after cellular stress, e.g. in C3H10T1/2 cells in response to anisomycin or UV [41] or in several cell lines in response to γ-irradiation, genotoxic compounds or during premature senescence [[42,43], reviewed in [6]].

We have also shown that sustained activation of p38 is required for contact-inhibition in murine and human fibroblasts [12]. Several mechanisms explaining p38-dependent cell cycle arrest have been described. For instance, p27^KIP1^, a well-known inhibitor of Cdk2 and Cdk4, is one important downstream target of p38 upon contact-inhibition [12,44]. The protein p27^KIP1 ^is also upregulated in response to genotoxic agents and here is supposed to be crucial for maintenance of cell cycle arrest [43]. However, we did not observe accumulation of p27^KIP1 ^in response to anisomycin or sorbitol (unpublished oberservation). It is also known that the Cdk inhibitors p21^WAF1/CIP1 ^and p16^INK4a ^mediate p38-dependent senescence, for instance in response to DNA-damaging agents and reactive oxygen species, which might be related to the role of p38 as a tumour suppressor [42,45-51]. Very recently, p38-dependent induction of p21 due to sorbitol-treatment has been described in nucleus pulposus intervertebral disc cells [52]. Whether p21^WAF1/CIP1 ^or p16^INK4a ^are upregulated in response to anisomycin or sorbitol in fibroblasts needs to be determined.

Moreover, we observed sustained phosphorylation of CREB. Two kinases are known to phosphorylate this transcription factor: MK2 [27,28] and MSK1 [26]. Since MK2 was also persistently activated in response to anisomycin, we conclude that MK2 at least partially contributes to phosphorylation of CREB. A possible involvement of MSK1 remains to be elucidated. Interestingly, cell cycle arrest due to sustained activation of the cAMP/CREB-pathway was also detected in prostate carcinoma cells, which were chronically exposed to pituitary adenylate-cyclase-activating polypeptide. In contrast, transient stimulation of cAMP/CREB induces proliferation [53]. Constitutive activation of CREB by bacterial toxins leads to G1-arrest in a murine macrophage cell line by induction of p27 and downregulation of cyclin D1 [54]. In accordance, cholera toxin, a potent inducer of cellular accumulation of cAMP and thereby phosphorylation of CREB, is able to cause G1 arrest by upregulation of p27, p21 and downregulation of cyclin D1 in rat and primary human glioma cells [55]. However, we could not reverse downregulation of cyclin D1 in response to sorbitol-exposure by SB203580 arguing that p38 is not the sole entity responsible for the decrease in cyclin D1. This is in line with the observation that arsenite-induced downregulation of cyclin D1 in NIH3T3 cells cannot be restored by SB203580 [8] and that cyclin D1 decrease can also be mediated by JNK [56]. Hence, the precise function of the p38-MK2-CREB axis in anisomycin- or sorbitol-induced cell cycle arrest remains to be determined.

Phosphorylation of CREB in response to mitogenic stimulation could not be blocked by SB203580, which is in line with previous observations that CREB phosphorylation in response to growth factors is mediated by the ERK pathway [26].

Interestingly, phosphorylation of ATF-2 was not inhibited by SB203580, although it has been identified to be an excellent substrate for p38 *in vitro *[25]. This observation is in perfect accordance with the work of Hazzalin and coworkers [41] and the work by Maher [13] ruling out involvement of p38 in phosphorylating endogenous ATF-2 in fibroblasts. Since phosphorylation of ATF-2 could be blocked by pharmacological inhibition of JNK, phosphorylation of ATF-2 is very likely dependent on JNK [57].

Persistent activation after anisomycin is consistent with the supposed mechanism of action: anisomycin inhibits protein synthesis hence blocking transcription of phosphatases. As a result, p38 dephosphorylation does not occur in the presence of anisomycin. Furthermore, dephosphorylation of the upstream acting MKK3/6 is inhibited thereby allowing prolonged activation of p38. A similar mechanism has been described for arsenite-induced JNK activation [58,59]. To gain more insight into upstream events regulating p38 activity in response to mitogens, we identified the involvement of small GTPases and made use of selective bacterial toxins. Clostridium sordellii Lethal Toxin (LT) inhibits Ras, Rac, and Rap function, Clostridium difficile Toxin B abolishes Rac, Cdc42, and Rho function, and Botulinus C3 exoenzyme, in our study used as C2IN-C3 fusion toxin displaying high cell permeability [60], selectively inhibits Rho function [29,30]. The observation, that ERK activation was selectively abolished only in the presence of Lethal Toxin indicates selectivity of the toxins. In control experiments with Botulinus C2 toxin, which ADP-ribosylates actin [29], we ruled out that the observed inhibitory effects of the toxins occurred unspecifically due to degradation of the actin cytoskeleton (unpublished observation). If anisomycin-induced p38 activation is due to its suppression of phosphatases (see above) it should be independent of Rho proteins. Indeed, blocking the activity of the small GTPases Rho, Rac, and Cdc42 by preincubation with Lethal Toxin or Toxin B, had no effect on anisomycin-induced p38 phosphorylation.

On the contrary, serum-induced p38 activation could be blocked by preincubation with Toxin B and Lethal Toxin. In view of the fact that C2IN-C3 had no effect on serum-induced p38 phosphorylation, the results strongly argue against an involvement of Rho and point to a potential role of Rac and/or Ras and Cdc42. Indeed, in overexpression studies, Rac and Cdc42 have been identified to mediate p38 activation [10,61,62]. More detailed analysis is required to identify which of the small GTPases is involved in p38-mediated control of proliferation.

## Conclusions

We present a novel hypothetical model for p38 function and propose a dual role of p38 activation in cell cycle control. Cellular stress by anisomycin leads to a sustained phosphorylation and activation of p38. One downstream substrate of p38 is MK2 which phosphorylates CREB. In contrast, after mitogenic stimulation p38 is only transiently phosphorylated, but promotes cyclin D1 expression. The cellular response is proliferation. Differential target activation by p38 downstream of mitogenic and stress signals might be related to the respective strength or duration of p38 activation or, alternatively, to additional cross-talk with parallel pathways - an issue which warrants further investigations.

## Materials and methods

### Cell culture

FH109 human embryonal lung fibroblasts [63] and NIH3T3 murine fibroblasts were routinely cultured in Dulbecco's Modified Eagle's Medium (DMEM) (PAA), supplemented with 10% fetal calf serum (FCS) (PAA), 4 mM glutamine, penicillin and streptomycin (each 100 U/ml). For experiments, FH109 or NIH3T3 cells were cultured in DMEM/0.2% FCS for 72 h and then treated with anisomycin (10 μg/ml) (Sigma) or FCS to a final concentration of 10% and harvested at different time points as described in the figure legends. Cells were pretreated with SB203580 (10 μΜ) (Calbiochem), or SP600125 (25 μM) (Enzo Life Sciences), control cells were exposed to 0.1% DMSO, the solvent used for the inhibitors.

### Measurement of DNA-synthesis

FH109 or NIH3T3 cells were seeded into microtiter plates at a density of 8 × 10^3^/well or 5 × 10^3^/well, respectively, and cultured for 72 h in DMEM/0.2% FCS. Cells were stimulated for 24 h by the addition of 10% FCS, PDGF- or bFGF (each 50 ng/ml, Cell Signaling). SB203580 was added 1 h before stimulation. DNA-synthesis was measured by [^3^H]thymidine-incorporation as described [64].

### Determination of cell number

Cells were washed, trypsinised and counted in a hemocytometer.

### Western blotting

Total cell extracts were prepared by lysing the cells in hot Laemmli sample buffer and protein concentration was determined according to [65]. Equal amounts of protein (20 - 50 μg protein/lane) were separated by SDS-PAGE (7.5 - 10%) and electroblotted overnight onto Immobilon membrane (Millipore). The membranes were blocked for 1 h with 5% low-fat milk-powder or 5% bovine serum albumin in TBS (50 mM Tris-HCl, pH 7.5, 150 mM NaCl) containing 0.05% Tween 20 and then incubated either for 1.5 h at room temperature with anti-cyclin D1-, anti-cyclin A- (1:1000, Santa Cruz), or overnight at 4°C with anti-phospho-pRb- (1:1000, Cell Signaling), anti-phospho-p38-, anti-CREB, anti-phospho-ATF-2-, anti-phospho-c-Jun-, or anti-phospho-MK2-antibody (1:1000-2000, Cell Signaling) followed by incubation with horseradish-peroxidase-conjugated secondary antibody and ECL-detection according to the manufacturer's instructions. The blots were stripped and reprobed with anti-p38α-, anti-phospho-CREB-, (1:1000, Cell Signaling), anti-ATF-2-, anti-PCNA-, anti-ERK-, or anti-c-Jun-antibody (1:1000, Santa Cruz) or anti-gelsolin-antibody (1:200, Santa Cruz) followed by ECL-detection.

### Transfection of siRNA

For transient transfection of p38α or control siRNA, 5 × 10^3 ^cells/well (96 well plate) were seeded and cultured for 24 h to reach 80-90% confluence. Transfection was performed in a total volume of 120 μl containing 8 pmol siRNA and 0.2 μl of Lipofectamine 2000 according to the manufacturer's instructions. After 24 h, medium was changed to DMEM/0.2% FCS and the cells cultured for another 24 h. Cells were then exposed for 24 h to PDGFβ or bFGF (see above) and incorporation of [^3^H]thymidine was determined. p38α siRNA (directed against murine p38α mRNA sequence [MGI:1346865]): 5'-GGAAUUCAAUGACGUGU AC-3'; control siRNA (directed against mRNA encoding the red fluorescence protein DsRed from the coral Discosoma) has been published previously [66].

## Competing interests

The authors declare that they have no competing interests.

## Authors' contributions

DF, CS and IS performed the experiments, CW and CD designed the experiments, CD wrote the paper, CW, FO and BO critically revised the manuscript. All authors read and approved the final manuscript.

## Supplementary Material

Additional file 1 **Effect of impairment of p38 function on mitogen-induced DNA synthesis**. Serum-starved FH109 cells were either not pretreated or preincubated for 1 h with SB203580 (10 μM). The cells were then stimulated with 10% FCS, PDGFβ, bFGF (each 50 ng/ml) or EGF (100 ng/ml) for 20 h and labelled with [3H]thymidine for additional 4 h. Incorporated radioactivity was determined by liquid scintillation spectrometry. Results are given as x-fold induction of [^3^H]thymidine incorporation compared to unstimulated, serum-starved cells and are the average ± Sx of a number of four for each run of treatment. The results represent one out of three independent experiments each leading to similar results. Click here for file

Additional file 2 **Specificity of SB203580-treatment and siRNA-mediated knock-down of p38**. (A, B) NIH3T3 cells were transiently transfected with siRNA directed against p38α or with control siRNA. 24 h after transfection, cells were serum-starved and cultured for another 24 h. Cells were then stimulated with PDGFβ (50 ng/ml) (A) or bFGF (50 ng/ml) (B) for the indicated time points. Western blot was performed using a monoclonal phospho-specific anti-p38-antibody. In parallel, the same samples were subjected to Western blot analysis using anti-p38-antibody. The blot was stripped and incubated with a monoclonal anti-phospho-ERK1/2-antibody. The blot was stripped again and subjected to immunoblotting using an ERK2-antibody to control equal loading. (C) Serum-starved NIH3T3 cells were treated with FCS in the absence or presence of SB203580 (10 μM) for the indicated time points. Western blot analysis was performed using anti-phospho-ERK1/2-antibody. Blots were stripped and reprobed with anti-ERK2-antibody to control equal loading. (D) NIH3T3 cells were not stimulated or stimulated with anisomycin in the absence or presence of SB203580 (10 μM), SP600125 (25 μM) or both inhibitors for the indicated time points. Western blot analysis was performed using a phospho-specific anti-c-Jun-antibody. Blots were stripped and reprobed with anti-c-Jun-antibody to control equal loading. Click here for file

Additional file 3 **Stimulation of p38 by mitogens and cellular stress**. Serum-starved FH109 cells were treated with FCS (A), or anisomycin (10 μg/ml) (B) and total cell extracts prepared at the indicated time points. Western Blot analysis was performed using a phospho-specific (T180/Y182) anti-p38-antibody. The blots were stripped and reprobed with an anti-p38-antibody to control equal loading. Data from a single experiment are shown. Similar results were obtained in three independent experiments. (C) Serum-starved FH109 cells were untreated or stimulated with FCS or anisomycin for 30 min. Kinase activity was measured after immunoprecipitation with a monoclonal phospho-specific anti-p38-antibody and ATF-2 fusion protein as substrate. Phosphorylation of ATF-2 was determined by Western blot analysis with a phospho-specific anti-ATF-2-antibody. Click here for file

Additional file 4 **Serum-starved NIH3T3 (A) or FH109 (B) cells were not pretreated or pretreated with SB203580 and then stimulated with FCS for the indicated time points**. Western blot analysis was performed using anti-cyclin D1- (A, B) or anti-cyclin A-antibodies (B). Click here for file

Additional file 5 **Analysis of p38 kinase mediated substrate phosphorylation**. Serum-starved FH109 cells were either not treated or pretreated with SB203580. (A, B) Cells were not stimulated, or stimulated either with FCS (A) or anisomycin (B) for 15 min. Total cell extracts were performed and subjected to Western blot analysis. Phosphorylation of CREB and ATF-2 was detected using phospho-specific antibodies, equal loading was controlled by anti-CREB- and anti-ATF-2-antibodies. The anti-phospho-CREB-antibody also recognizes phosphorylated ATF-1. ATF-2* = phosphorylated ATF-2 (C) Serum-starved FH109 cells were either not treated or stimulated with anisomycin and cell extracts prepared at the indicated time points. Western blot analysis for detection of CREB phosphorylation was performed as described above. Click here for file

Additional file 6 **Inhibition of small GTPases by bacterial toxins characteristically deregulates actin cytoskeleton and ERK1/2-phosphorylation**. Serum-starved NIH3T3 cells were either not pretreated or preincubated for 23 h with 400 ng/ml of C2IN-C3 (C3), for 2 h with 200 ng/ml of Lethal Toxin (LT), or for 2.5 h with 10 ng/ml of Toxin B (TB). (A) FITC-phalloidin staining to visualise activity of the bacterial toxins. As expected, exposure to C3 induces depolymerisation of actin stress fibres with little effect on lamellipodia or filopodia, TB strongly depolymerises the actin cytoskeleton [31], and LT causes rounding of cell bodies and disruption of actin stress fibres [32]. (B) Cells were either not stimulated or stimulated for 30 min with FCS. Western blot analysis was performed with anti-ERK1/2-antibody. As already shown by [32], LT inhibits phosphorylation of ERK1/2. ERK1*/ERK2* = phosphorylated ERK1/ERK2. Click here for file
