# Supplementary material for: Differential p38-dependent signalling in response to cellular stress and mitogenic stimulation in fibroblasts
Source: Cell Commun Signal. 2012 Mar 9;10:6. doi: 10.1186/1478-811X-10-6 (PMC3352310; doi:10.1186/1478-811X-10-6)
Supplement: Additional file 1 — Effect of impairment of p38 function on mitogen-induced DNA synthesis. Serum-starved FH109 cells were either not pretreated or preincubated for 1 h with SB203580 (10 μM). The cells were then stimulated with 10% FCS, PDGFβ, bFGF (each 50 ng/ml) or EGF (100 ng/ml) for 20 h and labelled with [3H]thymidine for additional 4 h. Incorporated radioactivity was determined by liquid scintillation spectrometry. Results are given as x-fold induction of [3H]thymidine incorporation compared to unstimulated, serum-starved cells and are the average ± Sx of a number of four for each run of treatment. The results represent one out of three independent experiments each leading to similar results. [file 1478-811X-10-6-S1.PDF]

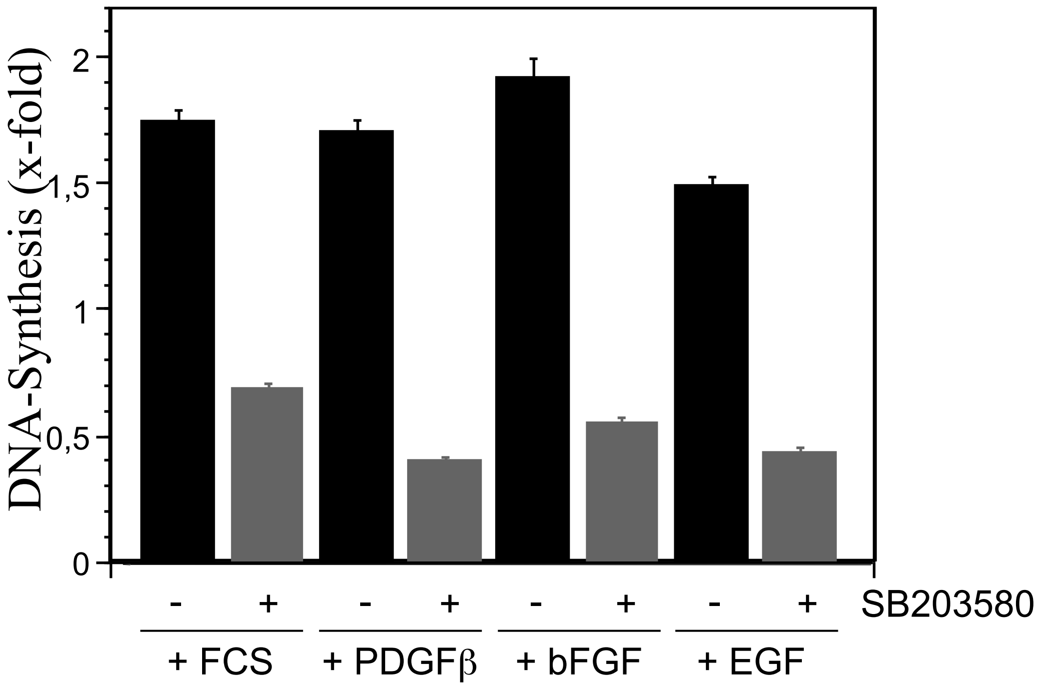

Additional file 1. Effect of impairment of p38 function on mitogen-induced DNA-synthesis.
Serum-starved FH109 cells were either not pretreated or preincubated for 1 h with SB203580 (10 μM). The cells were then stimulated with 10 % FCS, PDGFβ, bFGF (each 50 ng / ml) or EGF (100 ng / ml) for 20 h and labeled with [^{3}H]thymidine for additional 4 h. Incorporated radioactivity was determined by liquid scintillation spectrometry. Results are given as x-fold induction of [^{3}H]thymidine incorporation compared to unstimulated, serum-starved cells and are the average ± Sx of a number of four for each run of treatment. The results represent one out of three independent experiments each leading to similar results.
