# Supplementary material for: Differential p38-dependent signalling in response to cellular stress and mitogenic stimulation in fibroblasts
Source: Cell Commun Signal. 2012 Mar 9;10:6. doi: 10.1186/1478-811X-10-6 (PMC3352310; doi:10.1186/1478-811X-10-6)
Supplement: Additional file 2 — Specificity of SB203580-treatment and siRNA-mediated knock-down of p38. (A, B) NIH3T3 cells were transiently transfected with siRNA directed against p38α or with control siRNA. 24 h after transfection, cells were serum-starved and cultured for another 24 h. Cells were then stimulated with PDGFβ (50 ng/ml) (A) or bFGF (50 ng/ml) (B) for the indicated time points. Western blot was performed using a monoclonal phospho-specific anti-p38-antibody. In parallel, the same samples were subjected to Western blot analysis using anti-p38-antibody. The blot was stripped and incubated with a monoclonal anti-phospho-ERK1/2-antibody. The blot was stripped again and subjected to immunoblotting using an ERK2-antibody to control equal loading. (C) Serum-starved NIH3T3 cells were treated with FCS in the absence or presence of SB203580 (10 μM) for the indicated time points. Western blot analysis was performed using anti-phospho-ERK1/2-antibody. Blots were stripped and reprobed with anti-ERK2-antibody to control equal loading. (D) NIH3T3 cells were not stimulated or stimulated with anisomycin in the absence or presence of SB203580 (10 μM), SP600125 (25 μM) or both inhibitors for the indicated time points. Western blot analysis was performed using a phospho-specific anti-c-Jun-antibody. Blots were stripped and reprobed with anti-c-Jun-antibody to control equal loading. [file 1478-811X-10-6-S2.PDF]

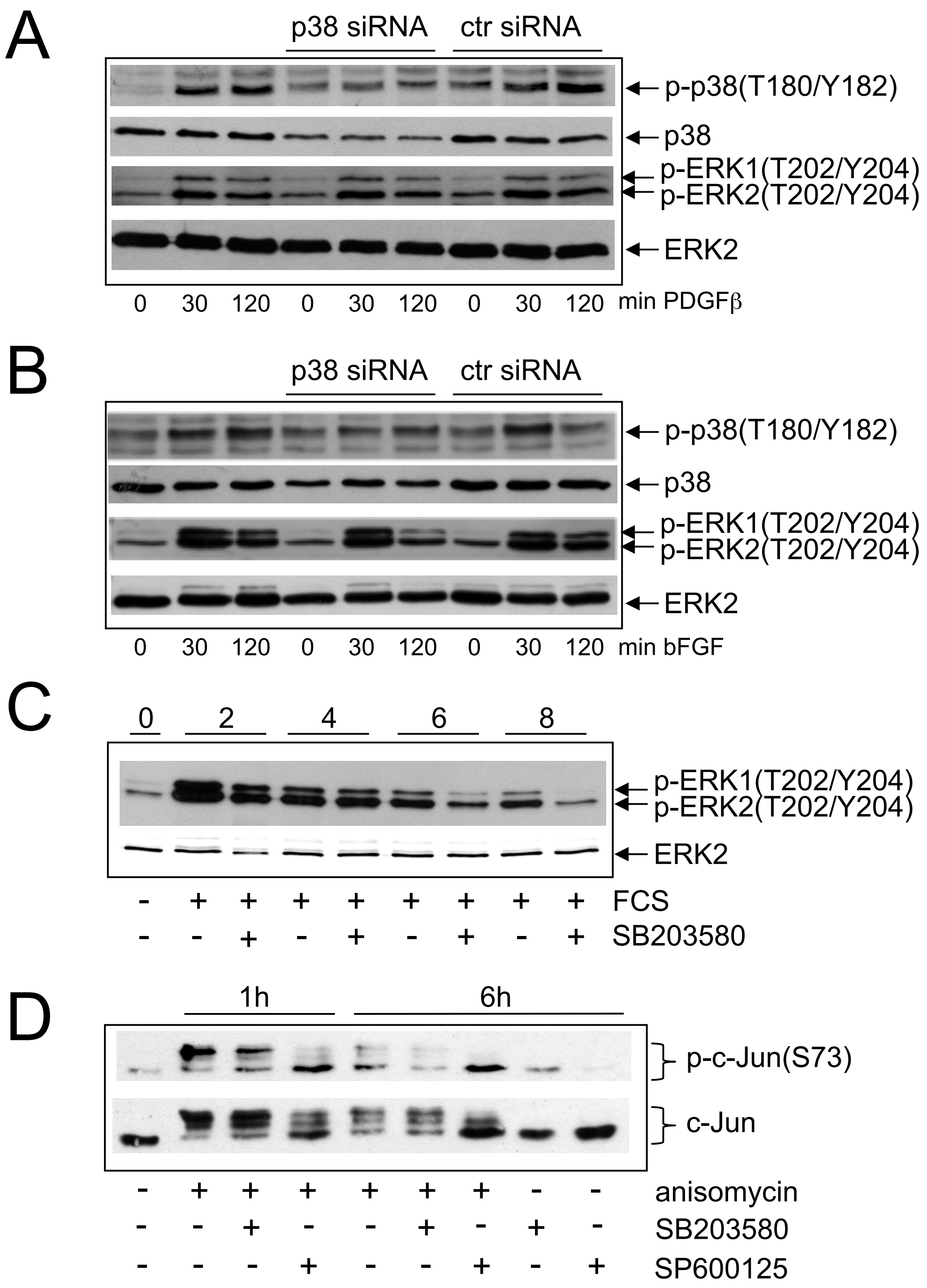

Additional file 2. Specificity of SB203580-treatment and siRNA-mediated knock-down of p38. (A, B) NIH3T3 cells were transiently transfected with siRNA directed against p38α or with control siRNA. 24 h after transfection, cells were serum-starved and cultured for another 24 h. Cells were then stimulated with PDGFβ (50 ng /ml) (A) or bFGF (50 ng/ml) (B) for the indicated time points. Western blot was performed using a monoclonal phospho-specific anti-p38-antibody. In parallel, the same samples were subjected to Western blot analysis using anti-p38-antibody. The blot was stripped and incubated with a monoclonal anti-phospho-ERK1/2-antibody. The blot was stripped again and subjected to immunoblotting using an ERK2-antibody to control equal loading. (C) Serum-starved NIH3T3 cells were treated with FCS in the absence or presence of SB203580 (10 μM) for the indicated time points. Western blot analysis was performed using anti-phospho-ERK1/2-antibody. Blots were stripped and reprobed with anti-ERK2-antibody to control equal loading. (D) NIH3T3 cells were not stimulated or stimulated with anisomycin in the absence or presence of SB203580 (10 μM), SP600125 (25 μM) or both inhibitors for the indicated time points. Western blot analysis was performed using a phospho-specific anti-c-Jun-antibody. Blots were stripped and reprobed with anti-c-Jun-antibody to control equal loading.
