# Supplementary material for: Differential p38-dependent signalling in response to cellular stress and mitogenic stimulation in fibroblasts
Source: Cell Commun Signal. 2012 Mar 9;10:6. doi: 10.1186/1478-811X-10-6 (PMC3352310; doi:10.1186/1478-811X-10-6)
Supplement: Additional file 3 — Stimulation of p38 by mitogens and cellular stress. Serum-starved FH109 cells were treated with FCS (A), or anisomycin (10 μg/ml) (B) and total cell extracts prepared at the indicated time points. Western Blot analysis was performed using a phospho-specific (T180/Y182) anti-p38-antibody. The blots were stripped and reprobed with an anti-p38-antibody to control equal loading. Data from a single experiment are shown. Similar results were obtained in three independent experiments. (C) Serum-starved FH109 cells were untreated or stimulated with FCS or anisomycin for 30 min. Kinase activity was measured after immunoprecipitation with a monoclonal phospho-specific anti-p38-antibody and ATF-2 fusion protein as substrate. Phosphorylation of ATF-2 was determined by Western blot analysis with a phospho-specific anti-ATF-2-antibody. [file 1478-811X-10-6-S3.PDF]

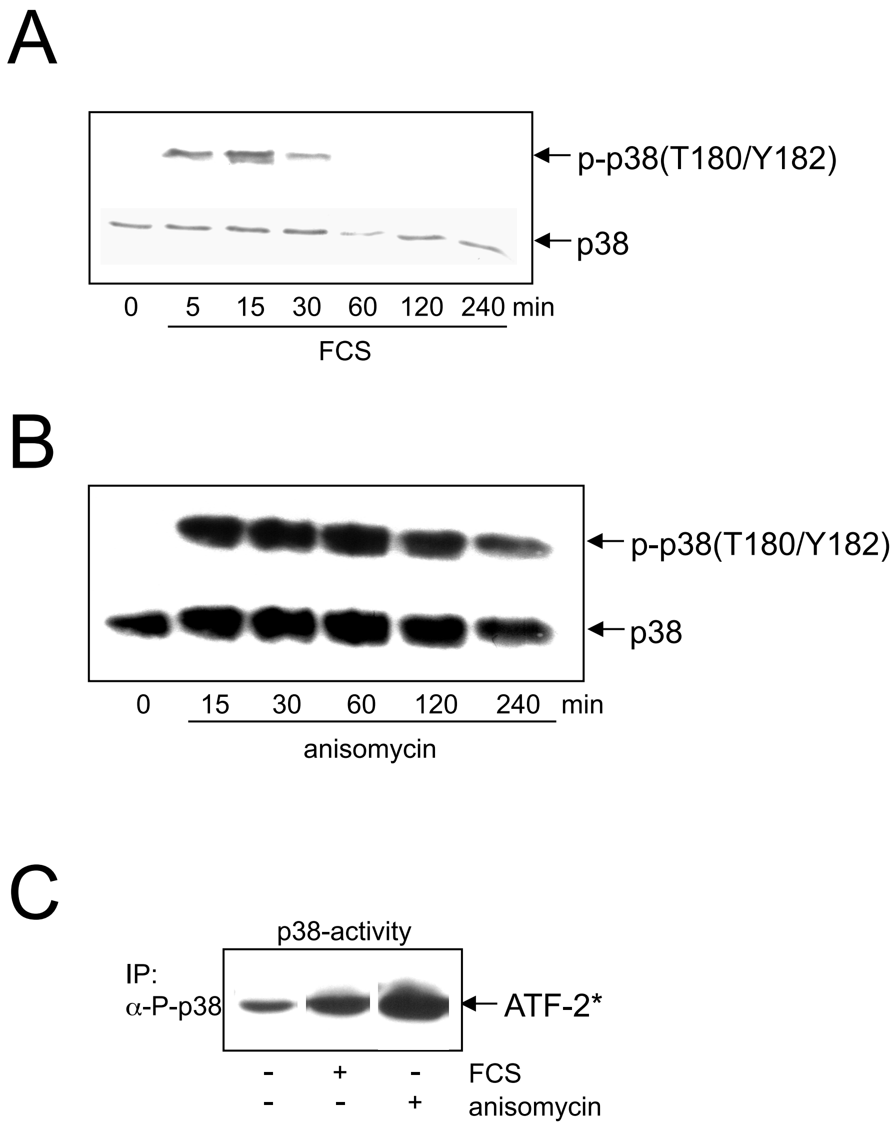

Additional file 3. Stimulation of p38 by mitogens and cellular stress.
Serum-starved FH109 cells were treated with FCS (A), or anisomycin (10 μg / ml) (B) and total cell extracts prepared at the indicated time points. Western Blot analysis was performed using a phospho-specific (T180/Y182) anti-p38 antibody. The blots were stripped and reprobed with an anti-p38-antibody to control equal loading. Data from a single experiment are shown. Similar results were obtained in three independent experiments. (C) Serum-starved FH109 cells were untreated (-) or stimulated with FCS or anisomycin for 30 min. Kinase activity was measured after immunoprecipitation with a monoclonal phospho-specific anti-p38-antibody and ATF-2 fusion protein as substrate. Phosphorylation of ATF-2 was determined by Western blot analysis with a phospho-specific anti-ATF-2-antibody.
