# Supplementary material for: Differential p38-dependent signalling in response to cellular stress and mitogenic stimulation in fibroblasts
Source: Cell Commun Signal. 2012 Mar 9;10:6. doi: 10.1186/1478-811X-10-6 (PMC3352310; doi:10.1186/1478-811X-10-6)
Supplement: Additional file 4 — Serum-starved NIH3T3 (A) or FH109 (B) cells were not pretreated or pretreated with SB203580 and then stimulated with FCS for the indicated time points. Western blot analysis was performed using anti-cyclin D1- (A, B) or anti-cyclin A-antibodies (B). [file 1478-811X-10-6-S4.PDF]

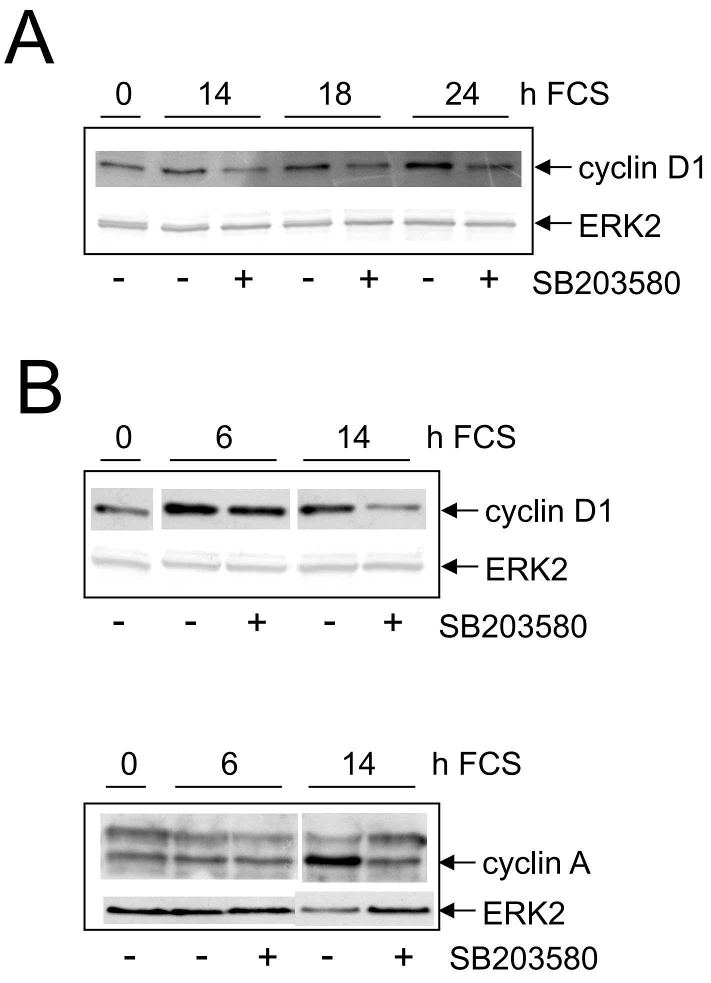

Additional file 4. Serum-starved NIH3T3 (A) or FH109 (B) cells were not pretreated or pretreated with SB203580 and then stimulated with FCS for the indicated time points. Western blot analysis was performed using anti-cyclin D1- (A, B) or anti-cyclin A-antibodies (B).
