# Supplementary material for: Differential p38-dependent signalling in response to cellular stress and mitogenic stimulation in fibroblasts
Source: Cell Commun Signal. 2012 Mar 9;10:6. doi: 10.1186/1478-811X-10-6 (PMC3352310; doi:10.1186/1478-811X-10-6)
Supplement: Additional file 5 — Analysis of p38 kinase mediated substrate phosphorylation. Serum-starved FH109 cells were either not treated or pretreated with SB203580. (A, B) Cells were not stimulated, or stimulated either with FCS (A) or anisomycin (B) for 15 min. Total cell extracts were performed and subjected to Western blot analysis. Phosphorylation of CREB and ATF-2 was detected using phospho-specific antibodies, equal loading was controlled by anti-CREB- and anti-ATF-2-antibodies. The anti-phospho-CREB-antibody also recognizes phosphorylated ATF-1. ATF-2* = phosphorylated ATF-2 (C) Serum-starved FH109 cells were either not treated or stimulated with anisomycin and cell extracts prepared at the indicated time points. Western blot analysis for detection of CREB phosphorylation was performed as described above. [file 1478-811X-10-6-S5.PDF]

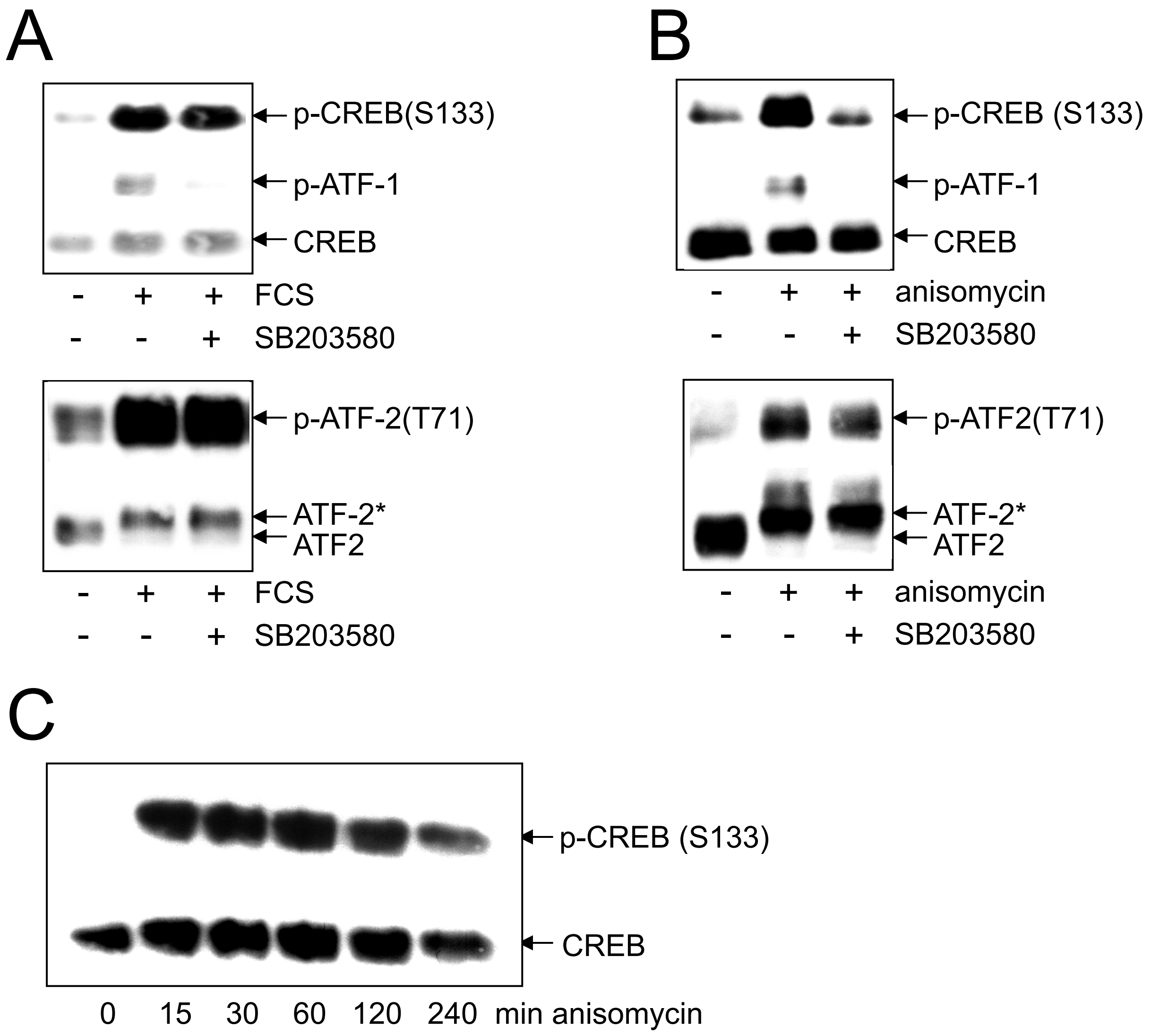

Additional file 5. Analysis of p38 kinase mediated substrate phosphorylation.
Serum-starved FH109 cells were either not treated or pretreated with SB203580. (A, B) Cells were not stimulated (-), or stimulated either with FCS (A) or anisomycin (B) for 15 min. Total cell extracts were performed and subjected to Western blot analysis. Phosphorylation of CREB and ATF-2 was detected using phospho-specific antibodies, equal loading was controlled by anti-CREB- and anti-ATF-2-antibodies. The anti-phospho-CREB-antibody also recognizes phosphorylated ATF-1. ATF-2* = phosphorylated ATF-2 (C) Serum-starved FH109 cells were either not treated (0) or stimulated with anisomycin and cell extracts prepared at the indicated time points. Western blot analysis for detection of CREB-phosphorylation was performed as described above.
