# Supplementary material for: Differential p38-dependent signalling in response to cellular stress and mitogenic stimulation in fibroblasts
Source: Cell Commun Signal. 2012 Mar 9;10:6. doi: 10.1186/1478-811X-10-6 (PMC3352310; doi:10.1186/1478-811X-10-6)
Supplement: Additional file 6 — Inhibition of small GTPases by bacterial toxins characteristically deregulates actin cytoskeleton and ERK1/2-phosphorylation. Serum-starved NIH3T3 cells were either not pretreated or preincubated for 23 h with 400 ng/ml of C2IN-C3 (C3), for 2 h with 200 ng/ml of Lethal Toxin (LT), or for 2.5 h with 10 ng/ml of Toxin B (TB). (A) FITC-phalloidin staining to visualise activity of the bacterial toxins. As expected, exposure to C3 induces depolymerisation of actin stress fibres with little effect on lamellipodia or filopodia, TB strongly depolymerises the actin cytoskeleton [31], and LT causes rounding of cell bodies and disruption of actin stress fibres [32]. (B) Cells were either not stimulated or stimulated for 30 min with FCS. Western blot analysis was performed with anti-ERK1/2-antibody. As already shown by [32], LT inhibits phosphorylation of ERK1/2. ERK1*/ERK2* = phosphorylated ERK1/ERK2. [file 1478-811X-10-6-S6.PDF]

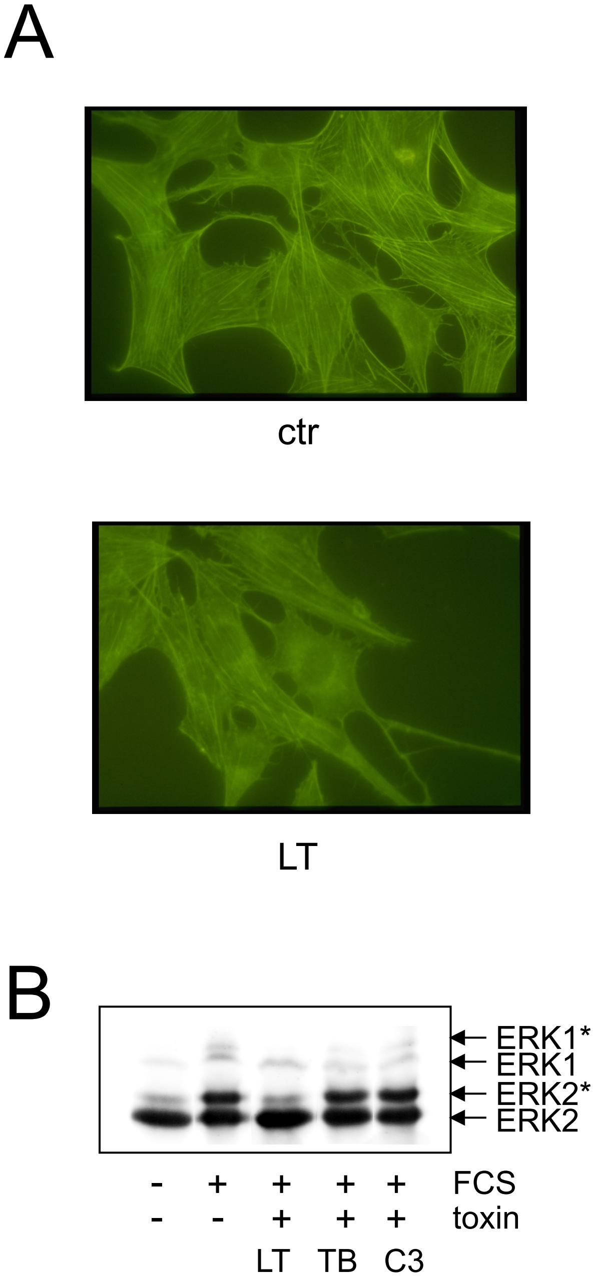


**Additional file 6. Inhibition of small GTPases by bacterial toxins characteristically deregulates actin cytoskeleton and ERK1/2-phosphorylation.**
Serum-starved NIH3T3 cells were either not pretreated (-) or preincubated for 23 h with 400 ng / ml of C2IN-C3 (C3), for 2 h with 200 ng / ml (2 h) of Lethal Toxin (LT), or for 2.5 h with 10 ng / ml of Toxin B (TB). (A) FITC-phalloidin staining to visualize activity of the bacterial toxins. As expected, exposure to C3 induces depolymerization of actin stress fibres with little effect on lamellipodia or filopodia, TB strongly depolymerizes the actin cytoskeleton [31], and LT causes rounding of cell bodies and disruption of actin stress fibres [32]. (B) Cells were either not stimulated (-) or stimulated for 30 min. with FCS. Western blot analysis was performed with anti-ERK1/2-antibody. As already shown by [32], LT inhibits phosphorylation of ERK1/2. ERK1* / ERK2* = phosphorylated ERK1/ERK2.
